# 乳酸化修饰在癌症中的研究进展

**DOI:** 10.3779/j.issn.1009-3419.2024.102.20

**Published:** 2024-06-20

**Authors:** Qicheng ZHANG, Limin CAO, Ke XU

**Affiliations:** 300052 天津，天津医科大学总医院，天津市肺癌研究所，天津市肺癌转移和肿瘤微环境重点实验室; Tianjin Key Laboratory of Lung Cancer Metastasis and Tumor Microenvironment, Tianjin Lung Cancer Institute, Tianjin Medical University General Hospital, Tianjin 300052, China

**Keywords:** 乳酸, 乳酸化修饰, 癌症, Lactate, Lactylation, Cancer

## Abstract

蛋白质的翻译后修饰（post-translational modifications, PTMs）可通过在前体蛋白的氨基酸残基上添加化学基团来改变其结构和功能，在信号转导、表观遗传和疾病等过程中发挥重要作用。乳酸化修饰（lactylation）是一种新发现的PTM，由于其与糖酵解代谢终产物乳酸密切相关，为理解细胞代谢重编程和表观遗传调控之间的联系提供了新视角。研究表明乳酸化修饰在肿瘤进展中发挥重要作用，并与临床不良预后相关。组蛋白的乳酸化异常可以影响肿瘤细胞和免疫细胞中的基因表达，从而进一步调控肿瘤的进展和免疫抑制。非组蛋白的乳酸化也可以调节肿瘤的增殖和耐药等过程。本文对乳酸化修饰领域的最新研究进展及其在肿瘤发生发展、微环境和免疫抑制中的作用和机制进行综述，并对其在肿瘤靶向治疗及联合免疫治疗方面的应用价值进行探讨。

## Abstract (en)

Post translational modifications (PTMs) can change the properties of a protein by covalent addition of functional groups to one or more amino acids, and influence almost all aspects of normal cell biology and pathogenesis. Lactylation is a novel identified PTM, and has been found in both histone and non-histone proteins. Since associated with the end product of glycolysis-- lactate, lactylation modification could provide a new perspective for understanding the relationship between metabolic reprogramming and epigenetic modifications. Accumulated evidences suggest that lactylation play important roles in tumor progression and links to poor prognosis in clinical studies. Histone lactylation can affect gene expression in tumor cells and immunological cells, further promoting tumor progression and immune suppression. Lactylation on non-histone proteins can also regulate tumor progression and drug resistance. In this review, we aimed to summarize the roles of lactylation in cancer progression, microenvironment interactions and immune suppression, try to identify new molecular targets for cancer therapy and provide a new direction for combined targeted therapy and immunotherapy.

蛋白质的翻译后修饰（post-translational modifications, PTMs）可通过在一个或多个氨基酸残基上添加化学基团来改变前体蛋白的生化特性，使其具有特定功能。常见的PTMs修饰包括磷酸化、乙酰化、泛素化、甲基化、糖基化等^[1]^。其中，发生在组蛋白上的PTMs可通过改变核小体间的接触或募集非组蛋白来发挥作用，是一类重要的表观遗传学修饰^[2]^。研究^[3]^发现PTMs与癌症的发生发展密切相关，参与调控包括增殖、凋亡、血管新生和侵袭转移等过程。

PTMs的修饰底物大多来源于细胞内的代谢过程^[4⇓-6]^。例如，乙酰辅酶A是细胞内的丙酮酸氧化脱羧及脂肪酸β氧化等过程产生的重要代谢产物，其是参与核心组蛋白乙酰化的底物^[7]^；S-腺苷甲硫氨酸在细胞内由ATP与甲硫氨酸在甲硫氨酸活化酶的作用下合成，其能够作为甲基供体调节蛋白的甲基化^[8]^。此外三羧酸循环产生的琥珀酰辅酶A可为蛋白质的琥珀酰化提供底物，由一氧化氮与游离的半胱氨酸残基反应形成的S-硝基硫醇可为S-亚硝基化提供底物等。乳酸是细胞内糖酵解反应的主要终产物，长期以来一直被视为代谢废物。肿瘤细胞的异常增殖和代谢重编程会消耗大量氧气和营养物质，导致肿瘤微环境（tumor microenvironment, TME）中的缺氧和营养缺乏，进一步引起肿瘤细胞及TME中乳酸水平的异常升高。近年来研究^[9]^发现，乳酸还是正常组织和肿瘤中多种信号通路的重要调节因子。TME中的乳酸，在包括肿瘤细胞、肿瘤相关成纤维细胞（cancer-associated fibroblasts, CAFs）、肿瘤相关巨噬细胞（tumor-associated macrophages, TAMs）和肿瘤浸润淋巴细胞（tumor infiltrating lymphocytes, TILs）等细胞间穿梭^[10]^，调控建立有利于肿瘤细胞生长和免疫逃逸的微环境^[11]^。

2019年，Zhang等^[12]^首次报道了乳酸诱导的一种新的PTM——乳酸化，他们发现组蛋白尾部的赖氨酸残基可以发生乳酸化。随后的研究证实乳酸化在多种癌症中普遍存在，并与恶性肿瘤的发展等过程密切相关。在这篇综述中，我们对乳酸化修饰，包括组蛋白和非组蛋白的乳酸化修饰在肿瘤的发生发展和微环境中细胞间相互作用等方面的研究进展进行综述，并探讨靶向乳酸代谢和乳酸化修饰相关酶进行肿瘤治疗的可能性，为开发新型的联合靶向治疗和免疫治疗方案提供思路。

## 1 肿瘤细胞的乳酸代谢及TME中的高乳酸水平

### 1.1 乳酸代谢

在正常情况下，细胞内的葡萄糖经过糖酵解和氧化磷酸化（oxidative phosphorylation, OXPHOS）最终产生大量ATP和CO_2_^[13]^。而在缺氧及运动、创伤、败血症和心力衰竭等应激条件下，细胞会进行无氧糖酵解，即糖酵解产生的丙酮酸由细胞质中的乳酸脱氢酶（lactate dehydrogenase, LDH）催化产生乳酸并释放少量能量^[9]^。Otto Warburg发现肿瘤细胞在存在足够氧气的情况下也会进行无氧糖酵解并分泌大量乳酸，并将肿瘤细胞代谢的这种特征称为有氧糖酵解或“Warburg效应”^[14,15]^。此外，肿瘤细胞还可以通过谷氨酰胺分解代谢产生乳酸^[16]^。虽然糖酵解和谷氨酰胺分解代谢产生的能量比OXPHOS少得多，但其这些过程产生的多种代谢中间体为包括核苷酸、嘌呤嘧啶、氨基酸、脂肪酸等大分子的合成提供了原材料，从而满足快速增殖的肿瘤细胞的需要^[17]^。

肿瘤细胞的“Warburg效应”是糖酵解相关酶和乳酸转运蛋白等基因改变和异常表达的结果。乳酸在细胞内外的运输由细胞表面的特定受体介导，主要包括单羧酸转运蛋白（monocarboxylate transporters, MCTs）、G蛋白偶联受体（G protein-coupled receptors, GPRs）（如GPR81）和存在于脑细胞中的羟基羧酸受体1（hydroxycarboxylic acid receptor 1, HCAR1）^[18]^。其中细胞内外乳酸的交换主要由MCT1和MCT4介导^[19]^。MCT1主要介导乳酸的摄入，而MCT4则主要介导细胞内乳酸的输出。研究^[20]^发现，通过抑制肿瘤细胞中MCT1和MCT4的表达来干扰乳酸代谢可以有效抑制肿瘤的生长。GPR81是细胞膜上的一种乳酸受体，其可在细胞外乳酸的刺激下直接介导细胞内多条信号通路的激活^[21]^。

缺氧诱导因子1α（hypoxia inducible factor-1α, HIF-1α）和c-Myc是肿瘤细胞维持高水平糖酵解的两个关键转录因子。它们可通过调控糖酵解中己糖激酶2（hexokinase 2, HK2）、丙酮酸激酶肌肉异构体2（pyruvate kinase muscle isoform 2, PKM2）以及LDHs的表达，抑制丙酮酸的线粒体代谢等多种机制调控乳酸的产生^[22]^，还可以调控肿瘤细胞中MCT1和MCT4的表达促进乳酸输出到TME以避免细胞内酸化^[20]^。此外，肿瘤细胞的“Warburg效应”还与哺乳动物雷帕霉素靶蛋白（mammalian target of rapamycin, mTOR）的活性有关。mTOR可以感知并整合各种环境信号并控制细胞生长和增殖，是细胞代谢网络中的关键调控节点。研究^[23]^发现mTOR通路的激活可提高HIF-1α和c-Myc的表达，进一步上调葡萄糖转运蛋白1（glucose transporter 1, GLUT1）、糖酵解相关酶及LDHA、MCT1和MCT4的表达，促进肿瘤细胞中乳酸的产生。

### 1.2 TME中的高乳酸水平和作用

肿瘤细胞可通过MCTs将有氧糖酵解产生的乳酸排到细胞外空间以避免其细胞内酸化。此外，研究^[24]^还发现TME中的其他细胞成分如CAFs等，也可以代谢产生并分泌乳酸。这些都会导致TME中乳酸水平的升高^[25]^。正常人血清中的乳酸浓度为1.5-3 mmol/L，而肿瘤患者血清中的乳酸浓度可升至10-30 mmol/L，在肿瘤内部甚至能达到50 mmol/L的水平^[26]^。研究^[27,28]^已证明TME中的高浓度乳酸与多种肿瘤的淋巴结或远端转移及低生存率等临床不良预后相关。TME中的乳酸可通过多种机制促进肿瘤细胞的增殖、血管生成、转移、耐药和免疫抑制^[29]^。此外，乳酸还与TME中的其他细胞成分，包括各种免疫细胞、CAFs、内皮细胞等的状态和功能密切相关，进一步调控免疫抑制、侵袭转移等过程^[30,31]^。

## 2 乳酸化修饰及相关蛋白

### 2.1 乳酸化修饰

组蛋白是染色体的重要组分，包括核心组蛋白（H2A、H2B、H3和H4）和连接组蛋白（H1和H5）。其带有正电荷，并与DNA结合一同形成核小体。组蛋白PTMs是表观遗传机制中的重要方式，通过共价修饰，不同的酰基可以连接到组蛋白的氨基酸残基上，进一步改变核小体的分子结构，影响各种效应物的募集或直接影响靶基因的转录^[32]^。组蛋白乳酸化修饰是2019年报道发现的一种新型组蛋白PTM。Zhang等^[12]^研究发现在缺氧或细菌感染引起内源性乳酸升高的情况下，细胞内的组蛋白乳酸化修饰水平显著提高；而抑制糖酵解中关键酶或LDH的酶活性则会导致组蛋白乳酸化修饰水平的下降，从而表明组蛋白乳酸化修饰与糖酵解和细胞内的乳酸水平直接相关。功能研究^[33]^发现，在巨噬细胞M1极化过程中，组蛋白乳酸化修饰可以以p53依赖和组蛋白乙酰转移酶p300介导的方式直接激活伤口愈合相关基因的表达以应对缺氧或炎症。目前已经鉴定出数十个组蛋白乳酸化修饰位点，主要发生在赖氨酸上，其中研究较多的有H3K18la、H4K5la、H4K18la等。此外，乳酸化修饰也可发生在非组蛋白中，通过影响蛋白质的结构和功能发挥多种作用^[34⇓-36]^。例如，Yang等^[37]^在肝癌细胞中鉴定出了9275个乳酸化修饰位点，其中9256个是在非组蛋白上；进一步的研究发现腺苷酸激酶2（adenylate kinase 2, AK2）K28位的乳酸化可通过影响p53通路的活性促进肿瘤细胞的增殖和转移。

### 2.2 乳酸化修饰相关蛋白

近年来对乳酸化修饰机制的研究揭示了其是一个动态可逆的过程，受到特定的乳酰基转移酶“writer”和去乳酰化酶“eraser”的共同调控，从修饰的赖氨酸残基上加上或去除乳酰基；进一步被称为“reader”的效应蛋白特异性识别和结合，影响下游信号通路并调控各种生物过程^[38]^。p300是一个经典的组蛋白乙酰转移酶（histone acetyltransferase, HAT），可以催化多种类型的蛋白质修饰并在恶性肿瘤发生发展过程中发挥重要作用^[39]^。Zhang等^[12]^研究发现p300也是乳酸化的writer蛋白，可以将L-乳酸转移到组蛋白上；过表达p300可显著提高细胞内的乳酸化修饰水平。组蛋白去乙酰化酶（histone deacetylase, HDAC）包括HDAC 1-11和沉默信息调节因子1-7（silent information regulator 1-7, SIRT1-7）两个家族，介导赖氨酸的去乙酰化过程。Moreno-Yruela等^[40]^研究发现HDACs也是组蛋白赖氨酸去乳酸化酶。体外研究显示HDAC 1-3可以显著降低H3K18la和H4K5la的水平，而SIRT1-3略微降低H3K18la和H4K5la的水平；在细胞中敲低HDAC1和HDAC3会导致H4K5la水平的增加，但总的乳酸化和H3K18la水平保持不变，且同时敲低HDAC 1-3会对H4K5la水平产生显著影响。体外和细胞内实验结果间的差异表明应该存在能使HDAC具有位点特异性去乳酸化活性的辅因子，但目前为止尚没有相关研究。总之，作为一种新型的PTM，目前对乳酸化修饰位点、修饰过程以及相关蛋白的认识仍然很有限，有待进一步开展深入研究。

### 2.3 乳酸化修饰与其他PTMs之间的关系

乳酸化修饰与其他PTMs之间的关系也为理解代谢和表观遗传过程之间的相互作用提供了新的方向。乙酰化和乳酸化修饰同受HATs和HDACs调节，且有研究显示两者之间存在密切的关系。Yang等^[41]^发现乳酸可以增加巨噬细胞中高迁移率族蛋白B1（high mobility group protein B1, HMGB1）的乳酸化和乙酰化修饰水平。乳酸化修饰与其他组蛋白修饰有关，如巴豆酰化和丁酰化。Dai等^[42]^报道发现乳酸化修饰和巴豆酰化对神经分化和细胞增殖过程具有协同作用。乳酸化修饰也可与丁酸介导的丁酰化有关。Xie等^[43]^发现丁酸可促进HeLa细胞中蛋白质乳酸化修饰水平的增加，而抑制HDACs可以显著降低这一效果。然而，目前关于乳酸化修饰与其他PTMs之间关系的研究尚处于初步阶段，有待进一步开展。图1为乳酸代谢和乳酸化修饰示意图。

**图1:**
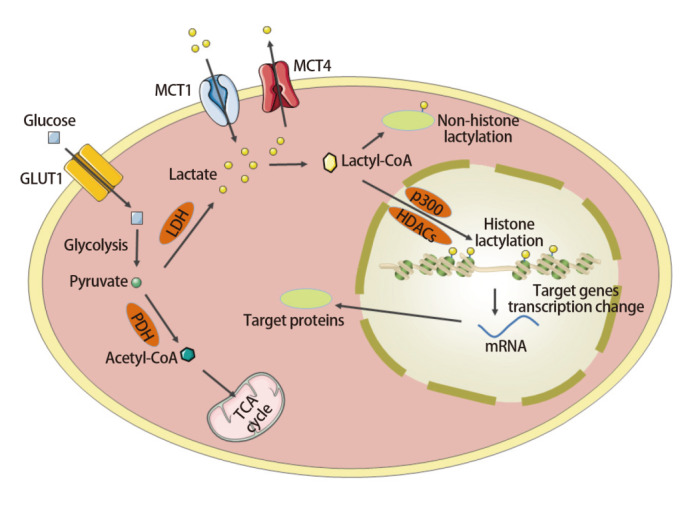
乳酸代谢和乳酸化修饰示意图。细胞内的乳酸可通过糖酵解产生的丙酮酸进一步经乳酸脱氢酶LDH催化产生，也可以通过单羧酸转运蛋白MCTs从细胞外提取。细胞内的乳酸可转化为乳酰辅酶A并进一步作为乳酸化修饰底物促进组蛋白和非组蛋白的乳酸化。

## 3 乳酸化修饰在肿瘤及TME中的作用和机制

### 3.1 乳酸化修饰及相关蛋白在肿瘤中的作用和机制

研究^[44,45]^表明，乳酸化修饰（包括组蛋白和非组蛋白）在多种肿瘤中发挥重要的促癌作用。在消化系统肿瘤（包括胃、肠和肝等）中，Yang等^[46]^发现胃癌组织中的乳酸化水平显著高于癌旁组织，且与胃癌的预后不良相关。另有研究^[47]^构建了胃癌组织中乳酸化修饰水平相关的预后模型，发现乳酸化评分与胃癌的总体生存和进展密切相关。Sun等^[48]^研究发现甲基转移酶METTL16的乳酸化修饰在胃癌细胞的铜死亡诱导过程中发挥重要作用。Miao等^[49]^发现低氧诱导的糖酵解可以通过促进β-连环蛋白的乳酸化修饰来增强其稳定性和表达，从而加剧结直肠癌细胞的恶性增殖。Zhou等^[50]^研究发现GPR37可以通过Hippo通路增强LDHA的表达和糖酵解，进一步提高组蛋白乳酸化修饰的水平，促进结直肠癌细胞的肝转移。Li等^[51]^近期报道发现乳酸可以通过组蛋白乳酸化修饰促进自噬增强子蛋白RUBCNL的表达，介导结直肠癌细胞对贝伐珠单抗的耐药性。Wang等^[52]^研究发现，肠道细菌来源的脂多糖（lipopolysaccharide, LPS）可通过诱导组蛋白乳酸化提高长非编码RNA--LINC00152的水平，进一步促进结直肠癌的侵袭和迁移。此外，Wu等^[53]^对肝癌中的乳酸化修饰进行组学分析后发现USP14和ABCF1蛋白上的特异性乳酸化位点可作为肝癌及其转移的诊断指标。Yang等^[37]^对乙型肝炎病毒相关的肝癌样本进行乳酸化修饰分析后发现乳酸化修饰会优先影响参与代谢途径的酶，并进一步证实乳酸化修饰可通过抑制AK2的功能促进肝癌细胞的增殖和转移。Cheng等^[54]^的分析表明乳酸化相关基因可作为肝癌临床治疗效果的生物标志物。在泌尿系统肿瘤（如肾脏、膀胱等）中，Yang等^[55]^发现组蛋白乳酸化修饰水平与肾透明细胞癌（clear cell renal cell carcinoma, ccRCC）患者较差的预后相关，机制研究显示组蛋白乳酸化可以通过激活血小板衍生生长因子受体β（platelet derived growth factor receptor β, PDGFRβ）的转录促进ccRCC的进展，靶向组蛋白乳酸化修饰可抑制ccRCC细胞在体内的增殖和转移。Xie等^[56]^在膀胱癌中的研究发现circXRN2-Hippo通路调节轴可通过抑制组蛋白乳酸化修饰和LCN2蛋白的表达调控肿瘤的进展。此外，Wang等^[57]^报道组蛋白乳酸化修饰可以促进带有BRAF突变的未分化甲状腺癌的增殖，联合使用乳酸代谢抑制剂和BRAF V600E抑制剂可以有效抑制甲状腺癌细胞的增殖。He等^[58]^发现，在前列腺癌和肺腺癌中，Numb/Parkin途径的缺失会导致代谢重编程，引起细胞内乳酸水平的显著增加，进而导致组蛋白乳酸化的增加和与神经内分泌功能相关的基因转录的激活。在眼黑色素瘤中，Yu等^[59]^研究发现组蛋白乳酸化修饰可以通过促进RNA N6-甲基腺苷（m^6^A）修饰识别蛋白YTHDF2的转录，进一步促进PER1和TP53 mRNA的降解，最终导致眼黑色素瘤细胞的增殖和迁移能力的提高。

应当注意的是，乳酸化修饰也可能发挥抑癌作用。Jiang等^[60]^对非小细胞肺癌（non-small cell lung cancer, NSCLC）的研究发现组蛋白乳酸化修饰水平的升高可导致糖酵解中血细胞激肽1（hemokinin-1, HK-1）和丙酮酸激酶M（pyruvate kinase M, PKM）水平的下降及三羧酸循环（tricarboxylic acid cycle, TCA）中的琥珀酸脱氢酶（succinate dehydrogenase, SDHA）和异柠檬酸脱氢酶3γ（isocitrate dehydrogenase 3γ, IDH3γ）水平的上升，进一步造成肿瘤细胞葡萄糖摄取和糖酵解的抑制以及细胞增殖和迁移能力的降低。Longhitano等^[61]^发现，乳酸可以通过增加乳酸诱导的组蛋白H3K18la修饰导致葡萄膜黑色素瘤（uveal melanoma, UM）细胞纯合子增加、细胞核增大和细胞停滞，进一步抑制UM的进展。

此外，乳酸化修饰相关酶在肿瘤中的作用近些年也有一些研究进展。Zu等^[62]^发现，组蛋白去乳酸化酶SIRT2可以抑制神经母细胞瘤细胞的增殖和迁移。Jin等^[63]^报道，去乳酸化酶SIRT3可以通过调控Cyclin E2的乳酸化修饰水平从而抑制肝癌细胞的增殖。

上述研究表明，揭示乳酸化修饰及相关蛋白在癌症进展和治疗中的作用和机制，可为肿瘤的临床诊断和靶向治疗提供新的标志物和靶点。同时要注意的是，乳酸化修饰及相关蛋白的具体功能可能因癌症而异，需要开展针对性研究对其具体作用及背后具体的分子机制进行探索。

### 3.2 乳酸化修饰在TME中的作用和机制

TME对肿瘤的发生发展至关重要。已有研究^[33]^发现，除了肿瘤细胞，乳酸及乳酸化修饰还可在TME中的其他细胞组分中发挥重要作用。免疫细胞是TME中的一大类细胞组分，在肿瘤细胞的免疫逃逸及免疫治疗等过程中发挥重要作用。已发现TME中的乳酸可以调节免疫细胞的代谢，抑制CD8^+^ T细胞、自然杀伤（natural killer, NK）细胞、树突状细胞等的增殖和功能，从而介导免疫逃逸^[21,64]^。巨噬细胞是TME中发挥重要功能的天然免疫细胞之一，其根据功能的不同分为促炎、抗癌的M1和抗炎、促癌的M2两种表型。Zhang等^[12]^研究发现，乳酸可以通过组蛋白乳酸化修饰诱导血管内皮生长因子等巨噬细胞M2表型相关蛋白的增加，促进巨噬细胞从M1表型向M2表型转化。Wang等^[65]^发现胆固醇代谢的关键分子前蛋白转化酶枯草溶菌素9（proprotein convertase subtilisin/kexin type 9, PCSK9）在结肠癌中高表达，敲低PCSK9会降低肿瘤细胞分泌的乳酸水平和巨噬细胞迁移抑制因子（macrophage migration inhibitory factor, MIF）的表达，进一步促进TAMs的M1极化并抑制其M2极化。Chaudagar等^[66]^在前列腺癌中的研究发现，磷脂酰肌醇-3激酶（phosphoinositide 3-kinase, PI3K）水平的降低会导致肿瘤细胞中乳酸合成的减少，进一步抑制TAMs中的组蛋白乳酸化修饰并增强其免疫功效。肿瘤浸润性髓系细胞（tumor infiltrating myeloid cells, TIMs）是一类与肿瘤免疫逃逸密切相关的天然免疫细胞，是肿瘤进展的关键调节者。Xiong等^[67]^在直肠癌中的研究发现TME中的乳酸可以通过引起TIMs中的组蛋白乳酸化修饰促进其中RNA N6-甲基腺苷（m^6^A）甲基转移酶METTL3的表达，进一步通过促进RNA m^6^A修饰并激活JAK1-STAT3信号通路发挥促癌作用。此外，TME中的调节性T细胞（regulatory T cell, Treg）在维持免疫抑制微环境（又称“冷”的TME）中发挥着至关重要的作用。Gu等^[68]^的研究发现，膜组织蛋白Moesin的乳酸化修饰可通过提高其与转化生长因子-β（transforming growth factor-β, TGF-β）受体的相互作用及激活SMAD3信号通路介导Treg细胞的产生，进一步促进肿瘤细胞的免疫逃逸；抑制Moesin的乳酸化修饰可以提高免疫治疗的疗效。Wang等^[69]^在恶性胸腔积液的“冷”TME中发现了一种特殊的FOXP3^+^ NKT样细胞，通过单细胞测序分析发现FOXP3^+^ NKT细胞高表达MCT和乳酸脱氢酶B，以摄取和利用乳酸，从而维持其免疫抑制功能。

以上这些发现揭示了乳酸及乳酸化修饰在TME中免疫细胞功能的调控中发挥重要作用，可为克服免疫抑制、提高肿瘤免疫治疗的效果提供新思路。此外，目前关于乳酸化修饰在TME中的其他具有重要功能的细胞组分如CAFs、内皮细胞等中的作用和机制尚未见报道，有待进一步开展。

## 4 针对乳酸化修饰的治疗

乳酸化修饰及其相关酶的研究^[70]^表明，靶向乳酸化修饰是抑制肿瘤进展和增强抗肿瘤作用的新选择，可以为抗肿瘤药物的开发提供了新的靶点。靶向乳酸化修饰可以从乳酸代谢、转运或乳酸化修饰等过程中的重要蛋白着手。目前已有的研究主要集中在乳酸代谢和转运相关蛋白的抑制剂上。

### 4.1 乳酸代谢相关抑制剂

抑制细胞内乳酸代谢过程中关键酶的活性可以通过降低乳酸的水平有效抑制乳酸化修饰。LDHA是糖酵解过程中丙酮酸转化为乳酸的关键酶，且其水平的上调是多种恶性肿瘤患者预后不良的指标。目前已经确定了几种有效的LDH抑制剂，如Oxamate可以作为丙酮酸竞争性LDHA抑制剂发挥作用，抑制肿瘤细胞的增殖^[71]^。NADH竞争性LDHA抑制剂，包括棉酚（Gossypol，也称AT-101）、3-二羟基-6-甲基-7-（苯基甲基）-4-丙基萘-1-羧酸（FX11）和喹啉-3-磺酰胺等，也被证明可以抑制肿瘤细胞的增殖^[72⇓-74]^。Gallflavin可通过直接结合抑制LDHA的活性，并可以抑制肿瘤细胞中乳酸的产生和诱导凋亡^[75]^。然而，由于LDHA抑制剂普遍存在的非靶向效应及与其他细胞成分存在复杂的相互作用，盲目抑制LDHA活性可能会产生一些难以控制的副作用，从而使这类抑制剂的临床开发受到限制。二氯乙酸盐（dichloroacetate, DCA）是一种口服小分子药物，可通过抑制丙酮酸脱氢酶激酶（pyruvate dehydrogenase kinase, PDK）和促进糖酵解中的葡萄糖氧化，进一步激活线粒体凋亡，抑制肿瘤细胞的增殖，并在胶质母细胞瘤和晚期头颈鳞癌中显示出良好的疗效^[76⇓-78]^。然而，乳酸代谢相关抑制剂，除了会影响细胞内的乳酸化修饰水平之外，还会对细胞内的代谢反应产生更加广泛且复杂的影响。因此，如何提高此类抑制剂的特异性，降低其副作用，以及分析其对于具体乳酸化修饰的抑制效果，是未来将此类抑制剂开发用于靶向乳酸化修饰治疗所需要解决的问题。

### 4.2 乳酸转运蛋白抑制剂

除了在细胞内产生外，乳酸还可以通过MCTs在细胞间穿梭和转运，进一步参与各种细胞生理和病理过程的调节。肿瘤细胞的乳酸代谢依赖于MCT4介导的乳酸流出和MCT1介导的乳酸流入，靶向MCTs可能对传统化疗耐药的癌症治疗有益^[19]^。目前已发现多种MCTs抑制剂，包括α-氰基-4-羟基肉桂酸盐（α-cyano-4-hydroxycinnamate, CHC）、根皮素（phloretin）、氯苯磺酸汞（p-chloromercuribenzenesulphonate）、4,40-二异硫氰基二苯乙烯-2,20-二磺酸（4,40-diisothiocyanatostilbene-2,20-disulphonic acid）、山莨菪碱（lonidamine）、槲皮素（quercetin）等，对多种肿瘤具有治疗潜力^[79,80]^。其中，山莨菪碱（lonidamine）是一种三萜类抗肿瘤化合物，可以通过抑制组蛋白H3K9和H3K56位点的乳酸化修饰来抑制肝癌干细胞的致癌作用，并已被研究与标准化疗联合使用于治疗实体瘤^[81,82]^。此外，一些新发现的MCTs小分子抑制剂具有更高的选择性。AR-C1555858^[83]^、AZD3965^[83]^和BAY-8002^[84]^已显示出强大的MCT1抑制和免疫调节活性，且已完成的一项I期临床试验^[85]^验证了AZD3965在晚期实体恶性肿瘤和淋巴瘤患者中的安全性和有效性^[83⇓-85]^。未来，进一步确定MCTs在不同癌症和乳酸化修饰过程中发挥的具体作用，以及其结构基础和调控机制，会为开发出特异性、靶向性更强的MCTs抑制剂并用于临床提供重要依据。

### 4.3 靶向乳酸化修饰对肿瘤免疫治疗的作用

此外，乳酸化修饰在肿瘤的免疫治疗过程中发挥重要作用。程序性细胞死亡受体1（programmed cell death protein 1, PD-1）阻断免疫治疗的效果是由TME中表达PD-1的CD8^+ ^T细胞和Treg细胞激活的竞争来决定。Gu等^[68]^发现乳酸可以通过调节Treg细胞中MOESIN的乳酸化修饰来促进肿瘤进展，并且在对PD-1抗体治疗有效的肝细胞癌患者Treg细胞中的乳酸化水平较低。Kumagai等^[86]^研究发现，在高糖酵解水平的恶性肿瘤中，Treg细胞通过MCT1快速吸收TME中的乳酸，刺激活化人活化T细胞核因子1（nuclear factor of activated T cells 1, NFAT1）进入细胞核并增加PD-1的表达，而效应T细胞PD-1表达则被抑制，最终造成治疗的失败。乳酸还可以促进巨噬细胞和中性粒细胞上程序性细胞死亡配体1（programmed cell death ligand 1, PD-L1）的表达以诱导免疫抵抗^[87]^。Weng等^[88]^报道靶向MCT1/miR-34a/IL-6/IL-6R信号轴可以抑制三阴性乳腺癌中巨噬细胞的M2极化。临床前研究^[89]^表明，将MCT1抑制剂AZD3965与抗PD-1疗法相结合可以降低乳酸盐向TME的释放并增强抗肿瘤免疫。此外，抗PD-1药物和LDHA抑制剂的组合也比单独的抗PD-1药物具有更强的抗肿瘤作用。以上发现表明将乳酸化修饰的抑制剂与免疫疗法相结合可能为肿瘤的联合治疗提供新的策略。

以上研究都表明靶向乳酸代谢和乳酸化修饰正成为一种潜在的和前瞻性的治疗策略。因此，对乳酸化修饰及其调控位点的探索可以进一步寻找有效的癌症治疗靶点，并为联合治疗提供新的方向。然而，尽管越来越多的证据表明乳酸是抗肿瘤和肿瘤治疗增敏的靶点，但乳酸化修饰在其中的作用和机制仍有待进一步阐明。此外，目前干预乳酸化修饰的手段主要集中在对乳酸生成、转运、信号转导的抑制，其对乳酸化修饰的特异性和有效性都有待提高。继续探索和识别乳酸化修饰中特异性“writer”“eraser”或“reader”蛋白，是真正靶向乳酸化修饰并为肿瘤治疗提供新靶点的关键任务。

## 5 总结和展望

蛋白质的乳酸化修饰不仅为PTMs的研究开辟了一个新的领域，也为肿瘤、免疫等领域的研究开辟了新的方向。此外，组蛋白乳酸化修饰的发现扩展了我们对代谢过程和表观遗传学修饰间相互关系的理解。然而，目前对于肿瘤细胞摄取和利用乳酸的具体机制尚未阐明，对蛋白质乳酸化修饰、代谢重编程和免疫抑制之间的相互作用也缺乏全面的了解。进一步阐明这些方面的机制和作用具有重要意义，还可以推动新的抗肿瘤治疗方法的开发。

## References

[1] Wang H, Yang L, Liu M, et al. Protein post-translational modifications in the regulation of cancer hallmarks. Cancer Gene Ther, 2023, 30(4): 529-547. doi: 10.1038/s41417-022-00464-3 35393571

[2] Kouzarides T. Chromatin modifications and their function. Cell, 2007, 128(4): 693-705. doi: 10.1016/j.cell.2007.02.005 17320507

[3] Pan S, Chen R. Pathological implication of protein post-translational modifications in cancer. Mol Aspects Med, 2022, 86: 101097. doi: 10.1016/j.mam.2022.101097 35400524 PMC9378605

[4] Kaelin WG Jr, McKnight SL. Influence of metabolism on epigenetics and disease. Cell, 2013, 153(1): 56-69. doi: 10.1016/j.cell.2013.03.004 23540690 PMC3775362

[5] Janke R, Dodson AE, Rine J. Metabolism and epigenetics. Annu Rev Cell Dev Biol, 2015, 31: 473-496. doi: 10.1146/annurev-cellbio-100814-125544 26359776 PMC5091661

[6] Reid MA, Dai Z, Locasale JW. The impact of cellular metabolism on chromatin dynamics and epigenetics. Nat Cell Biol, 2017, 19(11): 1298-1306. doi: 10.1038/ncb3629 29058720 PMC5886854

[7] Sabari BR, Zhang D, Allis CD, et al. Metabolic regulation of gene expression through histone acylations. Nat Rev Mol Cell Biol, 2017, 18(2): 90-101. doi: 10.1038/nrm.2016.140 27924077 PMC5320945

[8] Guccione E, Richard S. The regulation, functions and clinical relevance of arginine methylation. Nat Rev Mol Cell Biol, 2019, 20(10): 642-657. doi: 10.1038/s41580-019-0155-x 31350521

[9] Brooks GA. The science and translation of lactate shuttle theory. Cell Metab, 2018, 27(4): 757-785. doi: 10.1016/j.cmet.2018.03.008 29617642

[10] Certo M, Tsai CH, Pucino V, et al. Lactate modulation of immune responses in inflammatory versus tumour microenvironments. Nat Rev Immunol, 2021, 21(3): 151-161. doi: 10.1038/s41577-020-0406-2 32839570

[11] Ngwa VM, Edwards DN, Philip M, et al. Microenvironmental metabolism regulates antitumor immunity. Cancer Res, 2019, 79(16): 4003-4008. doi: 10.1158/0008-5472.CAN-19-0617 31362930 PMC6697577

[12] Zhang D, Tang Z, Huang H, et al. Metabolic regulation of gene expression by histone lactylation. Nature, 2019, 574(7779): 575-580. doi: 10.1038/s41586-019-1678-1 31645732 PMC6818755

[13] Ashton TM, McKenna WG, Kunz-Schughart LA, et al. Oxidative phosphorylation as an emerging target in cancer therapy. Clin Cancer Res, 2018, 24(11): 2482-2490. doi: 10.1158/1078-0432.CCR-17-3070 29420223

[14] Warburg O. On the origin of cancer cells. Science, 1956, 123(3191): 309-314. doi: 10.1126/science.123.3191.309 13298683

[15] Vander Heiden MG, Cantley LC, Thompson CB. Understanding the Warburg effect: the metabolic requirements of cell proliferation. Science, 2009, 324(5930): 1029-1033. doi: 10.1126/science.1160809 19460998 PMC2849637

[16] DeBerardinis RJ, Mancuso A, Daikhin E, et al. Beyond aerobic glycolysis: transformed cells can engage in glutamine metabolism that exceeds the requirement for protein and nucleotide synthesis. Proc Natl Acad Sci U S A, 2007, 104(49): 19345-19350. doi: 10.1073/pnas.0709747104 18032601 PMC2148292

[17] Pavlova NN, Zhu J, Thompson CB. The hallmarks of cancer metabolism: Still emerging. Cell Metab, 2022, 34(3): 355-377. doi: 10.1016/j.cmet.2022.01.007 35123658 PMC8891094

[18] Hadzic A, Nguyen TD, Hosoyamada M, et al. The lactate receptor HCA(1) is present in the choroid plexus, the tela choroidea, and the neuroepithelial lining of the dorsal part of the third ventricle. Int J Mol Sci, 2020, 21(18): 6457. doi: 10.3390/ijms21186457 32899645 PMC7554735

[19] Payen VL, Mina E, Van Hee VF, et al. Monocarboxylate transporters in cancer. Mol Metab, 2020, 33: 48-66. doi: 10.1016/j.molmet.2019.07.006 31395464 PMC7056923

[20] Doherty JR, Cleveland JL. Targeting lactate metabolism for cancer therapeutics. J Clin Invest, 2013, 123(9): 3685-3692. doi: 10.1172/JCI69741 23999443 PMC3754272

[21] Brown TP, Ganapathy V. Lactate/GPR81 signaling and proton motive force in cancer: Role in angiogenesis, immune escape, nutrition, and Warburg phenomenon. Pharmacol Ther, 2020, 206: 107451. doi: 10.1016/j.pharmthera.2019.107451 31836453

[22] Gordan JD, Thompson CB, Simon MC. HIF and c-Myc: sibling rivals for control of cancer cell metabolism and proliferation. Cancer Cell, 2007, 12(2): 108-113. doi: 10.1016/j.ccr.2007.07.006 17692803 PMC3215289

[23] Mossmann D, Park S, Hall MN. mTOR signalling and cellular metabolism are mutual determinants in cancer. Nat Rev Cancer, 2018, 18(12): 744-757. doi: 10.1038/s41568-018-0074-8 30425336

[24] Lee M, Yoon JH. Metabolic interplay between glycolysis and mitochondrial oxidation: The reverse Warburg effect and its therapeutic implication. World J Biol Chem, 2015, 6(3): 148-161. doi: 10.4331/wjbc.v6.i3.148 26322173 PMC4549759

[25] Parks SK, Pouyssegur J. Targeting pH regulating proteins for cancer therapy - Progress and limitations. Semin Cancer Biol, 2017, 43: 66-73. doi: 10.1016/j.semcancer.2017.01.007 28137473

[26] Brizel DM, Schroeder T, Scher RL, et al. Elevated tumor lactate concentrations predict for an increased risk of metastases in head-and-neck cancer. Int J Radiat Oncol Biol Phys, 2001, 51(2): 349-353. doi: 10.1016/S0360-3016(01)01630-3 11567808

[27] Walenta S, Wetterling M, Lehrke M, et al. High lactate levels predict likelihood of metastases, tumor recurrence, and restricted patient survival in human cervical cancers. Cancer Res, 2000, 60(4): 916-921. 10706105

[28] Dhup S, Dadhich RK, Porporato PE, et al. Multiple biological activities of lactic acid in cancer: influences on tumor growth, angiogenesis and metastasis. Curr Pharm Des, 2012, 18(10): 1319-1330. doi: 10.2174/138161212799504902 22360558

[29] Perez-Tomas R, Perez-Guillen I. Lactate in the tumor microenvironment: an essential molecule in cancer progression and treatment. Cancers (Basel), 2020, 12(11): 3244. doi: 10.3390/cancers12113244 33153193 PMC7693872

[30] Wang ZH, Peng WB, Zhang P, et al. Lactate in the tumour microenvironment: From immune modulation to therapy. EBioMedicine, 2021, 73: 103627. doi: 10.1016/j.ebiom.2021.103627 34656878 PMC8524104

[31] Gao Y, Zhou H, Liu G, et al. Tumor microenvironment: lactic acid promotes tumor development. J Immunol Res, 2022, 2022: 3119375. doi: 10.1155/2022/3119375 35733921 PMC9207018

[32] Millan-Zambrano G, Burton A, Bannister AJ, et al. Histone post-translational modifications - cause and consequence of genome function. Nat Rev Genet, 2022, 23(9): 563-580. doi: 10.1038/s41576-022-00468-7 35338361

[33] Qu J, Li P, Sun Z. Histone lactylation regulates cancer progression by reshaping the tumor microenvironment. Front Immunol, 2023, 14: 1284344. doi: 10.3389/fimmu.2023.1284344 37965331 PMC10641494

[34] Zhang N, Jiang N, Yu L, et al. Protein lactylation critically regulates energy metabolism in the protozoan parasite Trypanosoma brucei. Front Cell Dev Biol, 2021, 9: 719720. doi: 10.3389/fcell.2021.719720 34722503 PMC8551762

[35] Gao M, Zhang N, Liang W. Systematic analysis of lysine lactylation in the plant fungal pathogen Botrytis cinerea. Front Microbiol, 2020, 11: 594743. doi: 10.3389/fmicb.2020.594743 33193272 PMC7649125

[36] Mao Y, Zhang J, Zhou Q, et al. Hypoxia induces mitochondrial protein lactylation to limit oxidative phosphorylation. Cell Res, 2024, 34(1): 13-30. doi: 10.1038/s41422-023-00864-6 38163844 PMC10770133

[37] Yang Z, Yan C, Ma J, et al. Lactylome analysis suggests lactylation-dependent mechanisms of metabolic adaptation in hepatocellular carcinoma. Nat Metab, 2023, 5(1): 61-79. doi: 10.1038/s42255-022-00710-w 36593272

[38] Wang JH, Mao L, Wang J, et al. Beyond metabolic waste: lysine lactylation and its potential roles in cancer progression and cell fate determination. Cell Oncol (Dordr), 2023, 46(3): 465-480. doi: 10.1007/s13402-023-00775-z 36656507 PMC12974629

[39] Zeng Q, Wang K, Zhao Y, et al. Effects of the acetyltransferase p 300 on tumour regulation from the novel perspective of posttranslational protein modification. Biomolecules, 2023, 13(3): 417. doi: 10.3390/biom13030417 36979352 PMC10046601

[40] Moreno-Yruela C, Zhang D, Wei W, et al. Class I histone deacetylases (HDAC1-3) are histone lysine delactylases. Sci Adv, 2022, 8(3): eabi6696. doi: 10.1126/sciadv.abi6696 PMC8769552 35044827

[41] Yang K, Fan M, Wang X, et al. Lactate promotes macrophage HMGB 1 lactylation, acetylation, and exosomal release in polymicrobial sepsis. Cell Death Differ, 2022, 29(1): 133-146. doi: 10.1038/s41418-021-00841-9 34363018 PMC8738735

[42] Dai SK, Liu PP, Li X, et al. Dynamic profiling and functional interpretation of histone lysine crotonylation and lactylation during neural development. Development, 2022, 149(14): dev200049. doi: 10.1242/dev.200049 35735108

[43] Xie Y, Hu H, Liu M, et al. The role and mechanism of histone lactylation in health and diseases. Front Genet, 2022, 13: 949252. doi: 10.3389/fgene.2022.949252 36081996 PMC9445422

[44] Su J, Zheng Z, Bian C, et al. Functions and mechanisms of lactylation in carcinogenesis and immunosuppression. Front Immunol, 2023, 14: 1253064. doi: 10.3389/fimmu.2023.1253064 37646027 PMC10461103

[45] Zhang Y, Peng Q, Zheng J, et al. The function and mechanism of lactate and lactylation in tumor metabolism and microenvironment. Genes Dis, 2023, 10(5): 2029-2037. doi: 10.1016/j.gendis.2022.10.006 37492749 PMC10363641

[46] Yang D, Yin J, Shan L, et al. Identification of lysine-lactylated substrates in gastric cancer cells. iScience, 2022, 25(7): 104630. doi: 10.1016/j.isci.2022.104630 35800753 PMC9253728

[47] Yang H, Zou X, Yang S, et al. Identification of lactylation related model to predict prognostic, tumor infiltrating immunocytes and response of immunotherapy in gastric cancer. Front Immunol, 2023, 14: 1149989. doi: 10.3389/fimmu.2023.1149989 36936929 PMC10020516

[48] Sun L, Zhang Y, Yang B, et al. Lactylation of METTL 16 promotes cuproptosis via m(6)A-modification on FDX1 mRNA in gastric cancer. Nat Commun, 2023, 14(1): 6523. doi: 10.1038/s41467-023-42025-8 37863889 PMC10589265

[49] Miao Z, Zhao X, Liu X. Hypoxia induced beta-catenin lactylation promotes the cell proliferation and stemness of colorectal cancer through the wnt signaling pathway. Exp Cell Res, 2023, 422(1): 113439. doi: 10.1016/j.yexcr.2022.113439 36464122

[50] Zhou J, Xu W, Wu Y, et al. GPR37 promotes colorectal cancer liver metastases by enhancing the glycolysis and histone lactylation via Hippo pathway. Oncogene, 2023, 42(45): 3319-3330. doi: 10.1038/s41388-023-02841-0 37749229

[51] Li W, Zhou C, Yu L, et al. Tumor-derived lactate promotes resistance to bevacizumab treatment by facilitating autophagy enhancer protein RUBCNL expression through histone H3 lysine 18 lactylation (H3K18la) in colorectal cancer. Autophagy, 2024, 20(1): 114-130. doi: 10.1080/15548627.2023.2249762 37615625 PMC10761097

[52] Wang J, Liu Z, Xu Y, et al. Enterobacterial LPS-inducible LINC 00152 is regulated by histone lactylation and promotes cancer cells invasion and migration. Front Cell Infect Microbiol, 2022, 12: 913815. doi: 10.3389/fcimb.2022.913815 35959377 PMC9359126

[53] Wu X. In-depth discovery of protein lactylation in hepatocellular carcinoma. Proteomics, 2023, 23(9): e2300003. doi: 10.1002/pmic.202300003 37138381

[54] Cheng Z, Huang H, Li M, et al. Lactylation-related gene signature effectively predicts prognosis and treatment responsiveness in hepatocellular carcinoma. Pharmaceuticals (Basel), 2023, 16(5): 644. doi: 10.3390/ph16050644 37242427 PMC10221268

[55] Yang J, Luo L, Zhao C, et al. A Positive feedback loop between inactive VHL-triggered histone lactylation and PDGFRbeta signaling drives clear cell renal cell carcinoma progression. Int J Biol Sci, 2022, 18(8): 3470-3483. doi: 10.7150/ijbs.73398 35637958 PMC9134910

[56] Xie B, Lin J, Chen X, et al. CircXRN2 suppresses tumor progression driven by histone lactylation through activating the Hippo pathway in human bladder cancer. Mol Cancer, 2023, 22(1): 151. doi: 10.1186/s12943-023-01856-1 37684641 PMC10486081

[57] Wang X, Ying T, Yuan J, et al. BRAFV600E restructures cellular lactylation to promote anaplastic thyroid cancer proliferation. Endocr Relat Cancer, 2023, 30(8): e220344. doi: 10.1530/ERC-22-0344 37184950

[58] He Y, Ji Z, Gong Y, et al. Numb/Parkin-directed mitochondrial fitness governs cancer cell fate via metabolic regulation of histone lactylation. Cell Rep, 2023, 42(2): 112033. doi: 10.1016/j.celrep.2023.112033 36724072

[59] Yu J, Chai P, Xie M, et al. Histone lactylation drives oncogenesis by facilitating m(6)A reader protein YTHDF2 expression in ocular melanoma. Genome Biol, 2021, 22(1): 85. doi: 10.1186/s13059-021-02308-z 33726814 PMC7962360

[60] Jiang J, Huang D, Jiang Y, et al. Lactate modulates cellular metabolism through histone lactylation-mediated gene expression in non-small cell lung cancer. Front Oncol, 2021, 11: 647559. doi: 10.3389/fonc.2021.647559 34150616 PMC8208031

[61] Longhitano L, Giallongo S, Orlando L, et al. Lactate rewrites the metabolic reprogramming of uveal melanoma cells and induces quiescence phenotype. Int J Mol Sci, 2022, 24(1): 24. doi: 10.3390/ijms24010024 36613471 PMC9820521

[62] Zu H, Li C, Dai C, et al. SIRT2 functions as a histone delactylase and inhibits the proliferation and migration of neuroblastoma cells. Cell Discov, 2022, 8(1): 54. doi: 10.1038/s41421-022-00398-y 35672301 PMC9174446

[63] Jin J, Bai L, Wang D, et al. SIRT3-dependent delactylation of cyclin E 2 prevents hepatocellular carcinoma growth. EMBO Rep, 2023, 24(5): e56052. doi: 10.15252/embr.202256052 PMC10157311 36896611

[64] Tu CE, Hu Y, Zhou P, et al. Lactate and TGF-beta antagonistically regulate inflammasome activation in the tumor microenvironment. J Cell Physiol, 2021, 236(6): 4528-4537. doi: 10.1002/jcp.30169 33230810

[65] Wang L, Li S, Luo H, et al. PCSK9 promotes the progression and metastasis of colon cancer cells through regulation of EMT and PI3K/AKT signaling in tumor cells and phenotypic polarization of macrophages. J Exp Clin Cancer Res, 2022, 41(1): 303. doi: 10.1186/s13046-022-02477-0 36242053 PMC9563506

[66] Chaudagar K, Hieromnimon HM, Khurana R, et al. Reversal of lactate and PD-1-mediated macrophage immunosuppression controls growth of PTEN/p53-deficient prostate cancer. Clin Cancer Res, 2023, 29(10): 1952-1968. doi: 10.1158/1078-0432.CCR-22-3350 36862086 PMC10192075

[67] Xiong J, He J, Zhu J, et al. Lactylation-driven METTL3-mediated RNA m(6)A modification promotes immunosuppression of tumor-infiltrating myeloid cells. Mol Cell, 2022, 82(9): 1660-1677.e10. doi: 10.1016/j.molcel.2022.02.033 35320754

[68] Gu J, Zhou J, Chen Q, et al. Tumor metabolite lactate promotes tumorigenesis by modulating MOESIN lactylation and enhancing TGF-beta signaling in regulatory T cells. Cell Rep, 2022, 39(12): 110986. doi: 10.1016/j.celrep.2022.110986 35732125

[69] Wang ZH, Zhang P, Peng WB, et al. Altered phenotypic and metabolic characteristics of FOXP3(+)CD3(+)CD56(+) natural killer T (NKT)-like cells in human malignant pleural effusion. Oncoimmunology, 2023, 12(1): 2160558. doi: 10.1080/2162402X.2022.2160558 36567801 PMC9788685

[70] Fan H, Yang F, Xiao Z, et al. Lactylation: novel epigenetic regulatory and therapeutic opportunities. Am J Physiol Endocrinol Metab, 2023, 324(4): E330-E338. doi: 10.1152/ajpendo.00159.2022 36856188

[71] Zhao Z, Han F, Yang S, et al. Oxamate-mediated inhibition of lactate dehydrogenase induces protective autophagy in gastric cancer cells: involvement of the Akt-mTOR signaling pathway. Cancer Lett, 2015, 358(1): 17-26. doi: 10.1016/j.canlet.2014.11.046 25524555

[72] Van Poznak C, Seidman AD, Reidenberg MM, et al. Oral gossypol in the treatment of patients with refractory metastatic breast cancer: a phase I/II clinical trial. Breast Cancer Res Treat, 2001, 66(3): 239-248. doi: 10.1023/A:1010686204736 11510695

[73] Le A, Cooper CR, Gouw AM, et al. Inhibition of lactate dehydrogenase A induces oxidative stress and inhibits tumor progression. Proc Natl Acad Sci U S A, 2010, 107(5): 2037-2042. doi: 10.1073/pnas.0914433107 20133848 PMC2836706

[74] Billiard J, Dennison JB, Briand J, et al. Quinoline 3-sulfonamides inhibit lactate dehydrogenase A and reverse aerobic glycolysis in cancer cells. Cancer Metab, 2013, 1(1): 19. doi: 10.1186/2049-3002-1-19 24280423 PMC4178217

[75] Manerba M, Vettraino M, Fiume L, et al. Galloflavin (CAS 568-80-9): a novel inhibitor of lactate dehydrogenase. ChemMedChem, 2012, 7(2): 311-317. doi: 10.1002/cmdc.201100471 22052811

[76] Michelakis ED, Webster L, Mackey JR. Dichloroacetate (DCA) as a potential metabolic-targeting therapy for cancer. Br J Cancer, 2008, 99(7): 989-994. doi: 10.1038/sj.bjc.6604554 18766181 PMC2567082

[77] Dunbar EM, Coats BS, Shroads AL, et al. Phase1 trial of dichloroacetate (DCA) in adults with recurrent malignant brain tumors. Invest New Drugs, 2014, 32(3): 452-464. doi: 10.1007/s10637-013-0047-4 24297161 PMC4455946

[78] Powell SF, Mazurczak M, Dib EG, et al. Phase II study of dichloroacetate, an inhibitor of pyruvate dehydrogenase, in combination with chemoradiotherapy for unresected, locally advanced head and neck squamous cell carcinoma. Invest New Drugs, 2022, 40(3): 622-633. doi: 10.1007/s10637-022-01235-5 35312941 PMC9106928

[79] Amorim R, Pinheiro C, Miranda-Goncalves V, et al. Monocarboxylate transport inhibition potentiates the cytotoxic effect of 5-fluorouracil in colorectal cancer cells. Cancer Lett, 2015, 365(1): 68-78. doi: 10.1016/j.canlet.2015.05.015 26021766

[80] Perez-Escuredo J, Van Hee VF, Sboarina M, et al. Monocarboxylate transporters in the brain and in cancer. Biochim Biophys Acta, 2016, 1863(10): 2481-2497. doi: 10.1016/j.bbamcr.2016.03.013 26993058 PMC4990061

[81] Huang Y, Sun G, Sun X, et al. The potential of lonidamine in combination with chemotherapy and physical therapy in cancer treatment. Cancers (Basel), 2020, 12(11): 3332. doi: 10.3390/cancers12113332 33187214 PMC7696079

[82] Pan L, Feng F, Wu J, et al. Demethylzeylasteral targets lactate by inhibiting histone lactylation to suppress the tumorigenicity of liver cancer stem cells. Pharmacol Res, 2022, 181: 106270. doi: 10.1016/j.phrs.2022.106270 35605812

[83] Guan X, Rodriguez-Cruz V, Morris ME. Cellular uptake of MCT1 inhibitors AR-C155858 and AZD3965 and their effects on MCT-mediated transport of L-lactate in murine 4T1 breast tumor cancer cells. AAPS J, 2019, 21(2): 13. doi: 10.1208/s12248-018-0279-5 30617815 PMC6466617

[84] Quanz M, Bender E, Kopitz C, et al. Preclinical efficacy of the novel monocarboxylate transporter 1 inhibitor BAY-8002 and associated markers of resistance. Mol Cancer Ther, 2018, 17(11): 2285-2296. doi: 10.1158/1535-7163.MCT-17-1253 30115664

[85] Halford S, Veal GJ, Wedge SR, et al. A phase I dose-escalation study of AZD3965, an oral monocarboxylate transporter 1 inhibitor, in patients with advanced cancer. Clin Cancer Res, 2023, 29(8): 1429-1439. doi: 10.1158/1078-0432.CCR-22-2263 36652553 PMC7614436

[86] Kumagai S, Koyama S, Itahashi K, et al. Lactic acid promotes PD-1 expression in regulatory T cells in highly glycolytic tumor microenvironments. Cancer Cell, 2022, 40(2): 201-218.e9. doi: 10.1016/j.ccell.2022.01.001 35090594

[87] Deng H, Kan A, Lyu N, et al. Tumor-derived lactate inhibit the efficacy of lenvatinib through regulating PD-L 1 expression on neutrophil in hepatocellular carcinoma. J Immunother Cancer, 2021, 9(6): e002305. doi: 10.1136/jitc-2020-002305 PMC8231064 34168004

[88] Weng YS, Tseng HY, Chen YA, et al. MCT-1/miR-34a/IL-6/IL-6R signaling axis promotes EMT progression, cancer stemness and M2 macrophage polarization in triple-negative breast cancer. Mol Cancer, 2019, 18(1): 42. doi: 10.1186/s12943-019-0988-0 30885232 PMC6421700

[89] Huang T, Feng Q, Wang Z, et al. Tumor-targeted inhibition of monocarboxylate transporter 1 improves T-cell immunotherapy of solid tumors. Adv Healthc Mater, 2021, 10(4): e2000549. doi: 10.1002/adhm.202000549 PMC7674253 32431046

